# Reconciling Extent of Resection and Residual Tumor Volume in Glioblastoma: Sources of Discordance and a Proposed Reporting Framework

**DOI:** 10.7759/cureus.117312

**Published:** 2026-10-01

**Authors:** Prince Okong'o, Naomi Saro-Naenwi, Juliet Johnson, Sina Jamaria, Amira Salem, Venkata Kanishk Yannakula, Manju Rai

**Affiliations:** 1 Respiratory Medicine, Croydon Health Services NHS Trust, London, GBR; 2 General Medicine, Ministry of Health, Mater Dei Hospital, Msida, MLT; 3 Surgery, Royal London Hospital, London, GBR; 4 Medicine and Surgery, Gujarat Adani Institute of Medical Sciences (GAIMS), Bhuj, IND; 5 Neurosurgery, Salford Royal NHS Foundation Trust, Salford, GBR; 6 Surgery, Kasturba Medical College, Manipal, Manipal, IND; 7 Biotechnology, Shri Venkateshwara University, Gajraula, IND

**Keywords:** extent of resection, glioblastoma, idh-wildtype, methodological heterogeneity, neuro-oncology surgery, postoperative mri, rano-resect classification, residual tumor volume, standardized reporting, volumetric segmentation

## Abstract

Extent of resection (EOR) and residual tumor volume (RV) are among the few modifiable prognostic factors in glioblastoma (GBM), yet their interpretation remains complicated by substantial methodological heterogeneity. This narrative review synthesizes contemporary literature on EOR and RV as prognostic metrics in adult IDH-wild-type GBM, drawing on a structured search of PubMed/MEDLINE, Embase, Scopus, and Web of Science (January 2000-March 2026), and proposes a standardized reporting framework to improve cross-study comparability. Although both metrics are consistently associated with survival, apparent disagreement between EOR- and RV-based studies largely reflects methodological rather than genuine biological discordance. Key sources of heterogeneity include inconsistent terminology (18 distinct terms identified across six decades of literature, only about half with explicit thresholds), variable postoperative MRI timing, differing segmentation methods, inconsistent definitions of contrast-enhancing versus non-enhancing tumor compartments, evolving WHO molecular classification, and inadequate adjustment for confounding by indication, tumor location, and treatment factors. The Response Assessment in Neuro-Oncology (RANO)-resect classification represents an important advance toward standardization and has undergone independent revalidation, but definitional inconsistency persists more broadly. Building on these findings, this review proposes an author-derived, ten-domain reporting framework spanning study population, patient and tumor characteristics, surgical approach, imaging acquisition, segmentation methodology, EOR/RV calculation, molecular classification, confounder adjustment, statistical methods, and outcome reporting, intended to complement rather than replace existing standards such as Strengthening the Reporting of Observational Studies in Epidemiology (STROBE) and Transparent Reporting of a Multivariable Prediction Model for Individual Prognosis or Diagnosis (TRIPOD). This framework has not undergone formal Delphi consensus or prospective validation and should be regarded as a preliminary proposal. Standardized, transparent reporting, potentially supported by emerging AI-based segmentation and reporting tools, may help reconcile apparently conflicting findings and strengthen future evidence synthesis regarding surgical cytoreduction in GBM.

## Introduction and background

Glioblastoma (GBM) is the most aggressive primary malignant brain tumor in adults and, under the 2021 WHO classification, is defined predominantly by an isocitrate dehydrogenase (IDH)-wildtype molecular profile with characteristic molecular alterations and grade 4 behavior [1]. Despite advances in molecular classification and multimodal therapy, prognosis remains poor. Maximal safe surgical resection followed by radiotherapy and temozolomide remains a cornerstone of treatment, and the extent of surgical cytoreduction is one of the few potentially modifiable factors consistently associated with survival [2,3].

Extent of resection (EOR) has traditionally been used to quantify surgical cytoreduction, usually as the percentage of preoperative tumor volume removed. Increasing EOR has been associated with longer survival, while residual tumor volume (RV) provides an alternative measure based on the absolute amount of tumor remaining after surgery [4,5]. These measures are related but not equivalent: an identical percentage EOR can leave markedly different residual tumor burdens depending on initial tumor size. Contemporary evidence therefore increasingly supports considering EOR and RV as complementary rather than interchangeable measures of surgical cytoreduction.

However, substantial methodological heterogeneity complicates interpretation of either metric. The Response Assessment in Neuro-Oncology (RANO) resect group established volumetric categories incorporating residual contrast-enhancing (CE) tumor and, for supramaximal resection, residual non-CE tumor, providing an important step toward standardized EOR reporting [6]. Subsequent evidence has strengthened the clinical relevance of this approach. A 2025 meta-analysis of 31 studies involving 26,167 patients with newly diagnosed IDH-wild-type GBM demonstrated progressively better survival with increasing EOR and reported an additional survival advantage associated with supramaximal resection [7]. A 2026 population-based validation study further evaluated the RANO categories in an unselected real-world cohort, supporting their applicability beyond highly selected research populations [8]. At the same time, a 2025 systematic analysis of six decades of GBM literature identified 18 different terms for EOR and found that only about half of studies specifying a resection category also provided explicit thresholds, underscoring persistent definitional inconsistency [9].

RV introduces a related but distinct set of challenges. Absolute residual-volume thresholds vary considerably between studies, while postoperative measurements are influenced by MRI timing, acquisition parameters, segmentation methodology, and postoperative enhancement. Recent work has further suggested that an optimal RV threshold may vary by molecular context, emphasizing that residual disease burden cannot be interpreted independently of tumor biology [10]. The 2026 EANS-EANO guidelines now recognize complete removal of CE tumor, when safely achievable, as the recommended surgical goal for newly diagnosed IDH-wild-type GBM, while also emphasizing the neurological, cognitive, and quality-of-life consequences of surgical decisions [11]. Because the survival benefit of more radical resection must be weighed against these functional costs, EOR and RV thresholds derived from cohorts that exclude or censor patients with severe postoperative deficits, early death, or failure to receive adjuvant therapy may overstate the benefit of aggressive cytoreduction.

These developments indicate that the central problem is no longer simply whether greater EOR or smaller RV predicts better survival. Instead, the apparent prognostic value of these metrics may depend on a chain of interrelated factors, including imaging acquisition, tumor segmentation, metric definition, molecular classification, surgical context, functional outcome, and statistical adjustment. Previous reviews by Wykes et al. and Ius et al. provided important syntheses of evidence linking EOR and RV to outcomes [12,13]. This review differs by focusing on how these methodological and biological layers interact to produce apparently discordant findings between EOR- and RV-based studies and by translating this analysis into a multidimensional framework for interpreting and reporting surgical cytoreduction studies. The objective is therefore not to replace existing RANO classifications but to identify the additional methodological and clinical information required to determine when EOR and RV measurements are genuinely comparable and when apparent differences are primarily attributable to differences in measurement or study design.

## Review

Methods

This study was a critical narrative review of contemporary literature evaluating EOR and RV as prognostic metrics in adult patients with newly diagnosed GBM. The objective was not to perform a formal systematic review or meta-analysis but to synthesize the principal evidence comparing EOR and RV, identify methodological and clinical factors contributing to apparently discordant prognostic findings, and develop a practical framework for standardized reporting of surgical and volumetric outcomes.

A structured literature search was conducted in PubMed/MEDLINE, Embase, Scopus, and Web of Science for publications available from January 2000 through March 2026. This window was chosen pragmatically to capture approximately the most recent 25 years of literature and includes foundational studies of resection and survival, such as Lacroix et al. [3]. Because temozolomide chemoradiotherapy became standard of care after 2005 [2], the search period spans both pre- and post-Stupp treatment eras. The treatment era was therefore treated as a recognized source of heterogeneity in the synthesis: findings from cohorts treated before the adoption of chemoradiotherapy were interpreted in that context and not considered directly comparable with contemporary cohorts. Search terms were combined using Boolean operators and included “glioblastoma”, “glioblastoma IDH-wildtype”, “extent of resection”, “residual tumor volume”, “residual tumor”, “gross total resection”, “subtotal resection”, “volumetric analysis”, “postoperative MRI”, “RANO resect”, “supramaximal resection”, “tumor segmentation”, “survival”, “overall survival”, “progression-free survival”, and related terms. Table 1 summarizes the databases, date range, principal search concepts, and supplementary search methods.

**Table 1:** Literature search strategy for the narrative review EOR: extent of resection, IDH: isocitrate dehydrogenase, MRI: magnetic resonance imaging, RANO: Response Assessment in Neuro-Oncology, RV: residual tumor volume, GBM: glioblastoma

Database/source	Search period	Principal search concepts	Search approach	Supplementary search
PubMed/MEDLINE	January 2000-March 2026	GBM; IDH-wild-type GBM; EOR; RV; residual tumor; gross total resection; subtotal resection; volumetric analysis; postoperative MRI; RANO resect; tumor segmentation; survival	Boolean combinations of disease-, surgical-, imaging-, volumetric-, and outcome-related terms	Screening of reference lists of key eligible studies and reviews
Embase	January 2000-March 2026	Same core concepts, including controlled vocabulary and free-text terms relevant to GBM, EOR, RV, imaging, and survival	Database-specific controlled vocabulary and free-text Boolean searches	Citation chaining and reference-list screening
Scopus	January 2000-March 2026	GBM; EOR; RV; postoperative MRI; volumetric assessment; segmentation; survival; molecular classification	Title/abstract/keyword searches using Boolean combinations	Backward reference screening of relevant publications
Web of Science	January 2000-March 2026	GBM; EOR; RV; postoperative imaging; volumetric analysis; prognosis	Topic-based Boolean search	Citation tracking and reference-list screening
Additional sources	Through March 2026	Consensus statements, classification systems, reporting guidelines, methodological studies, and highly relevant publications identified during screening	Manual identification from reference lists and citation tracking	Targeted retrieval of publications addressing methodological standardization and reporting

Reference lists of key cohort studies, consensus statements, classification papers, systematic reviews, and methodological publications were also manually screened to identify additional relevant studies not captured by the electronic search. Eligible literature comprised prospective and retrospective cohort studies evaluating volumetrically quantified EOR or RV in adult patients with newly diagnosed GBM, together with systematic reviews, meta-analyses, consensus statements, classification studies, and methodological publications addressing surgical assessment, postoperative imaging, volumetric segmentation, molecular classification, statistical methodology, or reporting standards. No minimum or maximum interval between surgery and postoperative imaging was applied as an eligibility criterion. Because the objective was to characterize sources of heterogeneity between EOR- and RV-based studies, studies were retained irrespective of imaging timing. Timing was not systematically extracted for every study and is discussed where reported. Values from studies with late or unstated timing were therefore not regarded as directly comparable with those from early imaging and were not reclassified. Studies focused exclusively on recurrent GBM, pediatric populations, non-volumetric surgical outcomes, or unrelated glioma subtypes were generally excluded. Publications meeting these predefined clinical and methodological relevance criteria were retained for the narrative synthesis. Table 2 presents the principal inclusion and exclusion criteria.

**Table 2:** Inclusion and exclusion criteria EOR: extent of resection, IDH: isocitrate dehydrogenase, MRI: magnetic resonance imaging, OS: overall survival, PFS: progression-free survival, RV: residual tumor volume

Domain	Inclusion criteria	Exclusion criteria
Population	Adults (≥18 years) with newly diagnosed glioblastoma or contemporary molecularly defined IDH-wildtype glioblastoma	Pediatric patients and studies focused exclusively on recurrent or previously treated glioblastoma
Disease classification	Histologically or molecularly characterized glioblastoma; studies using contemporary or clearly specified historical diagnostic classifications	Studies involving mixed glioma populations in which glioblastoma-specific findings could not be distinguished
Surgical intervention	Studies evaluating surgical resection, biopsy, maximal safe resection, subtotal resection, gross/complete resection, or other surgical strategies in which EOR could be assessed	Studies with no evaluable surgical intervention or no information relevant to postoperative tumor burden
Surgical metric	Studies reporting EOR, RV, postoperative residual tumor burden, or volumetric measures that could be interpreted in relation to surgical extent	Studies reporting only qualitative surgical descriptions without an evaluable EOR or RV measure
Imaging/volumetry	Studies using postoperative MRI or other clearly described imaging-based methods to quantify residual tumor or assess EOR; timing of postoperative imaging was not an eligibility criterion	Studies lacking relevant postoperative imaging or volumetric assessment when such information was central to the study objective
Outcomes	Studies reporting OS, PFS, or other clinically relevant outcomes in relation to EOR or RV	Studies without relevant clinical or survival outcomes when prognostic assessment was the primary objective
Study design	Prospective or retrospective cohort studies, comparative studies, systematic reviews, meta-analyses, consensus statements, classification studies, and methodological studies relevant to EOR/RV assessment	Case reports, small case series without relevant volumetric or prognostic data, editorials, letters without substantive data, conference abstracts without sufficient information, and unrelated methodological publications
Methodological relevance	Publications addressing postoperative imaging, segmentation, molecular classification, statistical methodology, prognostic modeling, or reporting standards when directly relevant to interpretation of EOR/RV	Publications without a direct methodological, clinical, imaging, or prognostic relevance to EOR/RV assessment in glioblastoma
Language	Publications available in English	Publications for which an adequate English-language full text was unavailable
Publication period	Publications available from January 2000 through March 2026	Publications outside the predefined search period unless identified as historically important foundational literature through reference screening

Risk of bias in the principal prognostic cohort studies evaluating EOR and/or RV was assessed using the Quality In Prognosis Studies (QUIPS) tool (Appendices) [14]. The assessment considered six domains: study participation, study attrition, prognostic factor measurement, outcome measurement, study confounding, and statistical analysis and reporting. Two reviewers independently judged each domain as low, moderate, or high risk of bias based on the methodological characteristics reported in the original publication. Particular attention was given to patient selection and exclusions, completeness of follow-up, methods and timing of postoperative imaging and volumetric assessment, outcome ascertainment, adjustment for clinically relevant prognostic and treatment-related confounders, and the appropriateness and transparency of statistical analyses.

The literature was synthesized thematically according to the principal determinants of heterogeneity between EOR- and RV-based studies: mathematical definitions of surgical metrics; postoperative MRI timing and acquisition parameters; tumor compartment and segmentation methodology; evolution of WHO and molecular classification; anatomical and functional constraints on resection; confounding by indication and treatment-related factors; and statistical approaches to threshold selection and survival analysis. Particular attention was given to studies reporting absolute RV, standardized EOR classifications, and the relationship of these measures with overall survival (OS) and progression-free survival (PFS). A structured search and flow diagram (Figure 1) were used to improve transparency in the narrative review, not to imply that the review followed all PRISMA 2020 requirements.

**Figure 1:**
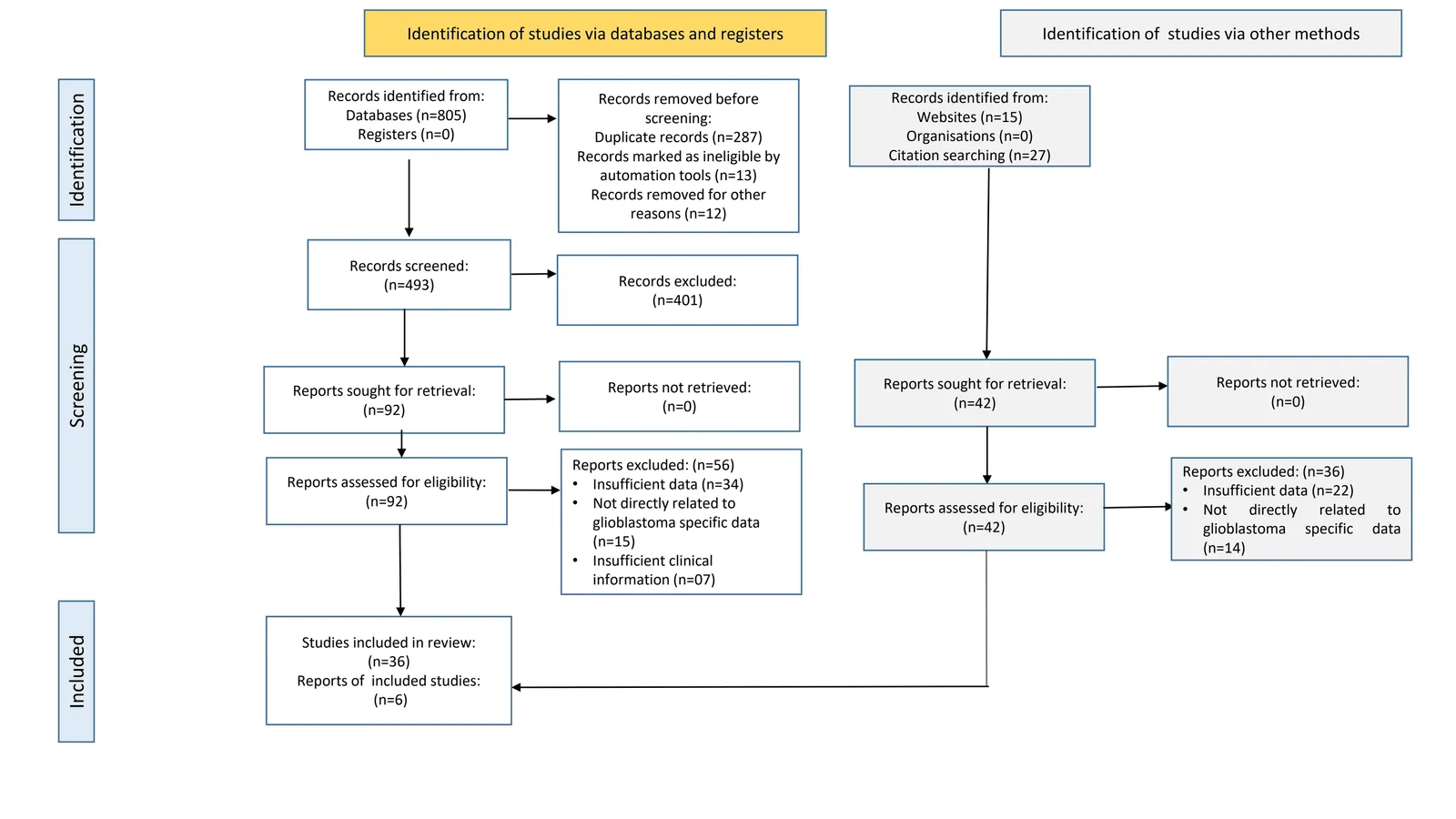
Flow diagram of the literature search and study selection process

The proposed standardized reporting framework was derived from recurrent methodological limitations identified across the reviewed literature. It was developed as an author-derived conceptual framework rather than through a formal Delphi process, multidisciplinary consensus meeting, prospective pilot study, or external validation exercise. The framework was designed to complement, rather than replace, established reporting standards and disease-specific classifications by specifying information required to interpret EOR and RV measurements reproducibly in GBM research. Its domains encompass imaging acquisition, tumor segmentation, surgical metric definitions, molecular and clinical characterization, confounder adjustment, statistical methodology, outcome reporting, and data transparency. The framework is therefore presented as a preliminary reporting proposal requiring prospective validation and broader stakeholder consensus before formal adoption.

EOR: evidence base and definitional problems

Evidence Supporting EOR as a Prognostic Marker

EOR has traditionally been the principal surgical metric used to evaluate cytoreduction in GBM. Early landmark studies demonstrated an association between greater resection and longer survival. Lacroix et al. reported progressively improved survival with increasing resection. Subsequently, Sanai et al. identified a quantitative relationship between EOR and survival, including an apparent threshold beyond which further removal was associated with additional benefit [3,15]. Subsequent systematic reviews and meta-analyses have generally confirmed the association between greater EOR and improved OS. However, differences in patient selection, tumor definition, imaging methodology, and EOR categorization have complicated interpretation [16,17].

A major advance was the development of the RANO resect classification, which sought to replace inconsistent descriptive terminology with objective volumetric categories [6]. Rather than relying solely on relative percentage reduction, the framework incorporates absolute postoperative RV and distinguishes maximal CE resection, submaximal resection, biopsy, and supramaximal resection. In the multicenter RANO resect validation study, lower residual CE volume was associated with better outcomes. Additionally, removal of non-CE tumor beyond the enhancing margin was associated with an additional survival benefit [18]. Importantly, the classification has subsequently undergone independent revalidation. A 2025 individual-patient-data meta-analysis reconstructed outcomes for 580 patients from three studies and confirmed significant prognostic separation across RANO resect categories, supporting the reproducibility of the classification beyond its original development cohort [8].

Terminological and Categorical Heterogeneity

The literature preceding and surrounding RANO resect demonstrates why EOR remains difficult to compare across studies. First, the terminology itself is inconsistent. Terms such as "gross-total", "complete", "total", "near-total", "subtotal", "partial", "maximal", and "supramaximal" resection have been used with different meanings. A systematic review of six decades of GBM research identified 18 different semantic terms for EOR and found that only approximately half of studies provided an explicit threshold defining their resection categories [9]. Thus, identical terminology does not necessarily represent an equivalent degree of tumor removal.

EOR may be defined categorically or continuously. Some studies analyze EOR as a continuous percentage of tumor removed, whereas others group patients by predetermined thresholds. These approaches answer different questions and can generate different estimates of prognostic effect. A continuous EOR variable preserves information but assumes an interpretable relationship across the resection range. In contrast, categorical thresholds can facilitate clinical interpretation but may introduce arbitrary cut points and information loss [3,15,17]. The problem is particularly relevant when cohorts contain predominantly high EOR values, because limited variability at the upper end of the distribution can make identification of a universal threshold statistically unstable.

The prognostic relationships of EOR and RV with survival should ideally be evaluated using continuous rather than purely categorical representations. Prior studies using nonlinear accelerated failure-time and other flexible survival models have shown continuous relationships between EOR or RV and OS, suggesting that fixed thresholds may not fully capture the underlying prognostic association [19,20]. Future studies should therefore consider flexible nonlinear approaches, such as restricted cubic splines or other prespecified continuous functions, to evaluate potential inflection points without imposing arbitrary step-function cutoffs. Importantly, studies should report baseline tumor volume alongside percentage EOR and consider it in model specification because the same percentage EOR can correspond to substantially different absolute RV [21].

Volumetric Definitions and Residual Tumor Burden

The underlying volumetric definition is not uniform. Percentage EOR requires both preoperative and postoperative tumor volumes, but the anatomical component being measured may differ. Studies may quantify only CE tumor on postcontrast T1-weighted MRI, incorporate non-CE abnormalities on T2/FLAIR imaging, or combine these measurements. This distinction becomes particularly consequential when considering supramaximal resection. The RANO resect framework explicitly separates CE and non-CE tumors and defines supramaximal resection according to residual non-CE volume after complete CE resection [18]. Conversely, studies restricted to CE tumor cannot be assumed to measure the same biological EOR as studies incorporating non-CE disease.

Absolute RV and percentage EOR can provide different prognostic information. Two patients may each undergo 90% EOR while retaining markedly different absolute tumor volumes if their preoperative tumors differ substantially in size. This supports reporting both relative reduction and absolute RV, rather than assuming percentage EOR alone adequately represents postoperative tumor burden. The RANO resect framework and contemporary studies evaluating residual disease as an independent prognostic variable reflect the importance of absolute RV [8,18].

Supramaximal Resection and Molecular Heterogeneity

Supramaximal resection adds another definitional layer. Recent multicenter work has incorporated supramaximal resection into prognostic models and demonstrated that its prognostic contribution can be considered alongside age, Karnofsky Performance Status (KPS), and O⁶-methylguanine-DNA methyltransferase (MGMT) promoter methylation [22]. Similarly, molecularly stratified analyses have shown that the relationship between resection of CE and non-CE tumor and survival is not necessarily uniform across clinical and molecular subgroups [23]. These findings suggest that the biological interpretation of an EOR threshold cannot be separated from the characteristics of the tumor being resected.

Molinaro et al. demonstrated molecular subgroup differences in the association between extended resection and survival [23], while a separate cohort of 326 patients with IDH-wild-type GBM found an association between ≥30% non-CE resection and longer survival particularly in MGMT-methylated tumors, but not in the unmethylated subgroup [24]. However, subgroup-specific significance does not establish a treatment interaction, and evidence for a TERT-specific modification of resection benefit remains limited.

Contemporary Evidence and Remaining Limitations

The contemporary evidence therefore supports EOR as an important prognostic marker, but not as a uniformly defined variable. The 2025 meta-analysis of 31 studies comprising 26,167 patients with newly diagnosed IDH-wild-type GBM provides strong contemporary evidence for a graded survival association with increasing EOR. Further, it supports the prognostic relevance of supramaximal resection [7]. A 2026 population-based study similarly found that RANO resect class 1 was associated with substantially longer median survival than lower resection classes in a real-world cohort, while also demonstrating the importance of perioperative neurological status [25]. These findings strengthen the evidence for aggressive but safe cytoreduction while simultaneously underscoring that the prognostic meaning of EOR depends on how resection is defined and measured.

Thus, the principal limitation of the EOR literature is no longer a lack of evidence that greater resection is associated with better outcomes. Rather, it reflects heterogeneity in what is called EOR, including terminology, percentage versus absolute thresholds, CE versus non-CE tumor assessment, categorical versus continuous analysis, and the handling of supramaximal resection. These differences should be considered when interpreting apparent discrepancies between studies and provide an essential basis for evaluating RV as a complementary postoperative metric.

RV: evidence base and measurement problems

Evidence Supporting RV as a Prognostic Metric

RV complements EOR as a measure of surgical cytoreduction by quantifying the absolute volume of tumor remaining after surgery rather than the proportion removed. This distinction matters clinically because percentage EOR depends mathematically on preoperative tumor volume: two patients with identical EOR may have substantially different postoperative tumor burdens. RV may therefore more directly represent the postoperative disease burden [4,5].

Chaichana et al. were among the first investigators to systematically evaluate both percentage EOR and RV. They demonstrated that postoperative RV was associated with survival and recurrence, with progressively smaller RV corresponding to more favorable outcomes [4]. Bette et al. subsequently confirmed the prognostic importance of postoperative tumor volume in 209 patients, finding that postoperative volume remained independently associated with survival after adjustment for age, KPS, MGMT promoter methylation, and adjuvant treatment [5]. Xing and Wang directly compared EOR and RV. They found that RV retained prognostic relevance in multivariable analyses, whereas EOR did not consistently remain an independent predictor, supporting the potential value of an absolute postoperative measure [26]. Similarly, Woo et al. evaluated RV in a multicenter cohort receiving temozolomide-based chemoradiotherapy and identified a postoperative volume threshold associated with survival, further supporting quantitative RV assessment as a clinically informative metric [27].

Postoperative Imaging and Small-Volume Measurement

Ellingson et al. provided additional validation using standardized postoperative volumetric assessment in a large clinical-trial population. Their analysis demonstrated that postoperative residual CE tumor volume was independently associated with OS after adjustment for important clinical and treatment-related factors, reinforcing the prognostic value of quantitatively measured residual disease rather than categorical descriptions of resection alone [28]. More recently, Filice et al. used subtraction MRI in 83 patients with IDH-wild-type GBM and found excellent interobserver agreement for RV (intraclass correlation coefficient, 0.91) [29]. RV remained significant alongside EOR, with a 1 cm³ threshold providing substantial prognostic discrimination and outperforming EOR-based modeling. This finding highlights how measurement methodology may influence the apparent prognostic value of RV. However, a 1 cm³ threshold should not be considered universally reliable, as postoperative blood products and cavity enhancement may confound very small-volume measurements. Although subtraction MRI may improve discrimination, evidence remains insufficient to mandate advanced imaging for all patients. Studies should therefore report imaging timing, technique, segmentation methods, and measurement reliability when evaluating small RV thresholds [29].

Postoperative Imaging Timing and Segmentation Methodology

Despite this evidence, RV remains a nonstandardized variable across the literature. A major potential source of heterogeneity is the timing of postoperative MRI. Early postoperative imaging is necessary to establish residual disease before postoperative reactive enhancement becomes prominent, but the optimal window is narrow. Current RANO-based practice generally favors MRI within 24-48 hours and no later than 72 hours after surgery because enhancement related to surgical manipulation can develop within 48-72 hours and persist thereafter [30]. Consequently, scans obtained at 24, 48, or 72 hours may not reflect equivalent postoperative tumor volumes. Blood products, ischemia, edema, and reactive enhancement may further complicate interpretation, particularly when quantifying small residual enhancing foci [30]. Even among RANO resect studies, the stated imaging window varies: Karschnia et al. imaged within 72 hours whenever possible [18], whereas Mendoza Mireles et al. imaged all patients within 48 hours [25]. Because imaging timing was not systematically extracted and is not uniformly reported, RV and EOR thresholds from studies with late or unreported timing are not treated in this review as equivalent to those from early-imaging cohorts. Thresholds are compared descriptively, and no threshold is proposed as a surgical target.

Another source of variability is segmentation methodology. RV can be estimated manually, semi-automatically, or through fully automated algorithms, and the choice affects reproducibility. Manual segmentation remains vulnerable to observer-dependent contouring decisions, particularly at the interface between the residual tumor and the postoperative cavity. Automated and semi-automated approaches can improve reproducibility but are dependent on the imaging sequences, preprocessing methods, software platform, and segmentation algorithm used. Thus, reporting an RV value without specifying how the tumor was segmented provides insufficient information to reproduce the measurement [28,31].

Anatomical Definition and Threshold Heterogeneity

The anatomical definition of RV differs between studies. Most classical RV analyses quantify residual CE tumor on postcontrast T1-weighted MRI. However, some investigations additionally quantify non-CE T2/FLAIR abnormalities, reflecting infiltrative tumor beyond the enhancing margin. Xing and Wang, for example, separately evaluated CE- and FLAIR-based tumor volumes, illustrating that the prognostic meaning of RV depends partly on which radiographic compartment is being measured [26]. These measures should not be treated as interchangeable: CE-RV primarily captures the postoperative enhancing tumor burden, whereas FLAIR-based measurements encompass a broader region that may include infiltrative tumor as well as edema, gliosis, or other treatment-related changes.

Another issue is variation in the RV thresholds used for prognostic classification. Studies have proposed different cutoffs, while others have modeled RV continuously. Woo et al. identified a clinically useful threshold in their multicenter cohort [27], whereas Chaichana et al. evaluated several residual-volume thresholds in relation to survival and recurrence [4]. Such thresholds should be interpreted as study-derived prognostic boundaries rather than universally validated surgical targets. A cutoff identified retrospectively in one cohort may not retain the same prognostic performance in a population with different baseline tumor volumes, molecular characteristics, imaging protocols, or treatment patterns.

Clinical and Biological Context of RV

RV is influenced by the same clinical and biological factors that affect EOR-based analyses. Age, KPS, tumor location, MGMT promoter methylation, adjuvant treatment, and molecular classification may be associated with both the achievable RV and subsequent survival [5,28,29]. More recent evidence therefore supports evaluating RV within contemporary molecularly defined cohorts rather than assuming that a single threshold applies across historically heterogeneous GBM populations.

Taken together, the evidence supports RV as a valuable and potentially more discriminative postoperative metric than percentage EOR, but its apparent precision should not be mistaken for methodological uniformity. The prognostic value of RV depends on when postoperative imaging is obtained, which tumor compartment is measured, how segmentation is performed, and how thresholds are derived and validated. These considerations explain why RV-based studies can reach different conclusions despite examining ostensibly the same clinical variable and provide the methodological basis for the subsequent analysis of why EOR- and RV-based findings may appear discordant.

Why EOR and RV conflict: sources of heterogeneity

Tumor Compartment and Imaging Heterogeneity

Apparent disagreement between studies using EOR and those using RV is substantially attributable to methodological heterogeneity rather than genuine biological discordance. Although both metrics describe postoperative tumor burden, they are not interchangeable: EOR expresses the proportion of the initial tumor removed, whereas RV represents the absolute amount remaining. Their prognostic estimates can therefore diverge when studies differ in imaging methodology, tumor definition, patient selection, or statistical adjustment.

The first source of heterogeneity is how the measured tumor compartment is defined. Many earlier EOR studies focused predominantly on CE tumor. In contrast, more recent RANO resect approaches distinguish residual CE tumor from non-CE tumor and incorporate both absolute RV and percentage reduction [32]. This distinction is important because non-CE abnormalities on T2/FLAIR imaging may represent infiltrative tumor but can also include edema, ischemia, gliosis, or surgically induced changes. Consequently, an EOR calculated from a CE tumor cannot be assumed to be directly comparable with an EOR incorporating non-CE disease. The greater interrater variability reported for non-CE segmentation further reinforces the importance of standardized definitions and centralized imaging assessment when analyzing this compartment [32].

Postoperative imaging timing represents a second major source of heterogeneity. RANO resect studies have generally emphasized early postoperative imaging, with MRI obtained within approximately 72 hours whenever possible, because later scans may contain reactive enhancement, contrast leakage, or early tumor progression that can be mistaken for residual disease [32]. Automated segmentation studies have similarly emphasized early postoperative imaging as a prerequisite for reliable classification of residual CE tumor [31]. Therefore, a study using MRI at 24 hours, another at 48-72 hours, and a third using a substantially later postoperative examination may be assigning different biological and radiological meanings to the same nominal RV or EOR category.

Studies also differ in how they address reactive enhancement, blood products, and ischemia. Karschnia et al. imaged within 72 hours whenever possible and excluded surgically induced edema or ischemia from non-CE volumes [18]; Mendoza Mireles et al. imaged all patients within 48 hours and included diffusion-weighted sequences [25]; and Ellingson et al. used T1 subtraction to correct for blood products in the resection cavity [28]. When postoperative imaging is delayed, or its timing is not stated, published data cannot reliably separate residual enhancement from reactive enhancement, contrast leakage, or early progression, and RV values from such studies should be interpreted as non-comparable with those from early imaging [33,34].

Segmentation and Volumetric Measurement

A third factor is the method used to quantify residual disease. Manual, semi-automated, and automated segmentation can produce different estimates, particularly when small residual enhancing foci are present at the resection margin. The RANO resect group showed generally favorable agreement for postoperative CE tumor volumetry but greater variability for non-CE tumor assessment, indicating that reproducibility depends on the compartment being measured [32]. More recent automated approaches may improve consistency and scalability, but performance still depends on acquisition protocols, preprocessing, segmentation algorithms, and validation cohorts [31]. Thus, a numerical RV without description of the segmentation method is insufficient to establish that two studies measured the same variable.

Tumor Size, Patient Selection, and Confounding

Tumor size provides another explanation for apparently conflicting EOR and RV findings. Because EOR is a ratio, it incorporates preoperative tumor volume into its calculation. A high percentage EOR may therefore coexist with a clinically substantial absolute residual tumor burden in a large tumor. In contrast, the same RV may represent a much larger proportion of a smaller tumor. This mathematical distinction can alter the apparent strength of association with survival. It may explain why studies based on absolute RV sometimes identify stronger prognostic relationships than studies using percentage EOR [4,5]. Consequently, the RANO resect framework places greater emphasis on absolute postoperative RV when categorizing resection [32].

Patient selection and confounding by indication are additional sources of apparent discordance. Achieving a very small RV depends not only on surgical performance but also on tumor location, involvement of eloquent structures, preoperative neurological status, tumor size, and the feasibility of safe cytoreduction. Patients in whom complete or near-complete resection is achievable may consequently have intrinsically more favorable clinical characteristics than patients with substantial residual disease [33,34]. Conversely, deliberately limiting resection because of functional risk can result in a larger RV without implying an inferior surgical strategy. Recent RANO resect analyses have attempted to address this problem through multivariable adjustment and propensity-score approaches incorporating clinical, molecular, anatomical, and treatment-related variables [35]. These approaches reduce, but cannot eliminate, the limitations inherent to retrospective observational data.

Molecular and Treatment-Related Heterogeneity

Molecular classification adds another layer of heterogeneity. Historical GBM cohorts were assembled using diagnostic criteria that predated the WHO 2021 classification, whereas contemporary cohorts increasingly restrict analysis to molecularly defined IDH-wild-type GBM [1,32]. Molecular characteristics, particularly MGMT promoter methylation, are independently associated with outcome and may be unevenly distributed between patients undergoing different degrees of resection. Consequently, an apparent survival advantage associated with high EOR or low RV may partly reflect differences in tumor biology if the analysis does not adequately incorporate molecular variables. Contemporary studies that adjust for MGMT status and other clinical variables provide a more appropriate basis for evaluating the independent prognostic contribution of postoperative tumor burden [35-36].

Treatment heterogeneity can similarly modify the observed relationship between postoperative tumor burden and survival. Differences in receipt and completion of radiotherapy and temozolomide, treatment delays, and subsequent therapies may influence survival independently of the amount of tumor remaining after surgery. The prognostic association of smaller RV has persisted in analyses incorporating treatment variables. However, the magnitude of this association should not be assumed to be identical across cohorts with different treatment patterns [35-36].

The treatment era is another source of variation: cohorts accrued before the widespread adoption of temozolomide chemoradiotherapy after 2005 [2] received different adjuvant treatment and were also diagnosed before widespread MGMT testing and the WHO 2016 and 2021 classifications, so their survival estimates cannot be assumed comparable with contemporary cohorts.

Selection, Survivorship, and Statistical Bias

Selection and survivorship bias represent another source of distortion in studies reporting "optimal" EOR or RV thresholds. Radical resection carries a risk of new neurological deficit, and patients who die perioperatively, develop severe deficits, or cannot receive adjuvant chemoradiotherapy are less likely to appear in cohorts that require completion of the Stupp protocol or a minimum survival. When such patients are excluded or censored, the adverse consequences of the resection trade-off are underrepresented, inflating the apparent benefit of aggressive cytoreduction. Requiring receipt of adjuvant therapy as an entry criterion also introduces immortal time bias [37]. The functional consequences are substantial: in one retrospective series, new postoperative deficits were associated with shorter survival even when they later resolved, and the survival benefit of achieving 95% EOR was abrogated in patients who developed a new deficit [38]. The timing of adjuvant therapy adds another layer. An analysis of RTOG trials found no survival decrement with delays of up to six weeks and unexpectedly longer survival in patients starting radiotherapy more than four weeks after surgery than in those starting within two weeks [39], while a national database analysis reported that the association between delay and survival differed by resection category [40]. Time to adjuvant therapy is therefore entangled with recovery, complications, and case selection and should be reported alongside volumetric measures rather than treated as a background covariate. Because postoperative deficits and treatment receipt lie on the causal pathway between resection and survival, we recommend that primary analyses include all patients undergoing surgery, with time zero defined as the date of surgery. Models adjusted for these variables (estimating a direct effect) or landmark analyses should be reported as secondary analyses.

Table 3 summarizes the principal methodological factors contributing to the apparent disagreement between EOR- and RV-based studies, along with recommended approaches to improve consistency.

**Table 3:** Principal sources of apparent heterogeneity underlying conflicting prognostic findings between EOR- and RV-based studies CE: contrast-enhancing, CI: confidence interval, EOR: extent of resection, IDH: isocitrate dehydrogenase, MGMT: O⁶-methylguanine-DNA methyltransferase, MRI: magnetic resonance imaging, RV: residual tumor volume, T2-FLAIR: T2-weighted fluid-attenuated inversion recovery, WHO: World Health Organization

Source of heterogeneity (representative evidence)	Influence on EOR studies	Influence on RV studies	Contribution to apparent disagreement	Recommended approach
Mathematical definition of surgical metrics (Lacroix et al. [3]; Chaichana et al. [4]; Bette et al. [5]; Filice et al. [29])	EOR expresses the proportion of preoperative tumor removed and is therefore a relative measure dependent on baseline tumor volume.	RV represents the absolute postoperative tumor burden and is independent of the initial tumor volume.	The same EOR can correspond to substantially different RVs in tumors of different baseline sizes; percentage-based and absolute-volume analyses may therefore identify different prognostic relationships.	Report both EOR (%) and absolute RV (cm³), together with preoperative and postoperative volumes and the calculation method. For prognostic analyses, preferably retain EOR and RV as continuous variables, and assess nonlinear associations using appropriate flexible modeling approaches where justified. Studies should report baseline tumor volume, postoperative RV, and percentage EOR concurrently, and should assess whether baseline tumor burden modifies the relationship between EOR and outcome. If clinically relevant thresholds are identified, their derivation, uncertainty, validation, and dependence on baseline tumor volume should be explicitly reported.
Imaging acquisition and postoperative timing (Ellingson et al. [28]; Karschnia et al. [18]; Luque et al. [31])	Inconsistent preoperative and postoperative imaging can alter the tumor volumes used to calculate EOR.	RV is particularly sensitive to postoperative MRI timing, blood products, reactive enhancement, ischemia, and surgical-cavity changes.	MRI obtained at different postoperative intervals may identify different apparent amounts of residual enhancement, making EOR and RV estimates incompletely comparable across studies.	Prespecify postoperative MRI timing, preferably within 24-48 hours and no later than 72 hours when feasible, and report field strength, sequences, contrast administration, and relevant acquisition parameters.
Tumor segmentation and volumetric methodology (Ellingson et al. [30]; Luque et al. [31])	EOR depends on accurate delineation of preoperative and postoperative tumor volumes.	RV is directly determined by postoperative segmentation and is therefore highly sensitive to segmentation strategy.	Manual, semi-automated, and automated methods, and differences in software or contouring conventions, can produce different measurements.	Specify segmentation method, software/platform, tumor compartment, reader characteristics, quality-control procedures, and interobserver agreement where available.
Tumor compartment and definition of residual disease (Karschnia et al. [6]; Karschnia et al. [18]; Molinaro et al. [23]; Filice et al. [29])	EOR may be calculated from CE tumor alone or may incorporate non-CE disease.	RV may refer exclusively to residual CE tumor or include non-CE/T2-FLAIR abnormalities.	CE-only and CE-plus-non-CE measurements represent different radiological and biological entities and should not be treated as equivalent endpoints.	Clearly define the tumor compartment used for EOR/RV calculation and report CE and non-CE components separately when analyzed.
Evolution of tumor classification (Louis et al. [1]; Karschnia et al. [6]; Weller et al. [32])	Historical EOR cohorts frequently included tumors classified under older diagnostic systems and therefore contained greater biological heterogeneity.	Historical RV cohorts are similarly affected by evolving diagnostic and molecular classification.	Differences between historical and contemporary cohorts may reflect changes in tumor classification and case mix rather than differences in the prognostic effect of surgical cytoreduction.	Report diagnostic classification and molecular status and, where possible, analyze contemporary WHO-defined molecular cohorts separately.
Molecular heterogeneity (Molinaro et al. [23]; Park et al. [22])	The association between higher EOR and survival may vary by molecular characteristics, including MGMT and other biomarkers.	The prognostic meaning of a given RV may similarly vary according to tumor biology.	Unequal distribution or incomplete reporting of molecular characteristics can produce different survival estimates across EOR- and RV-based cohorts.	Incorporate relevant molecular biomarkers into baseline reporting, multivariable analyses, and prespecified subgroup or interaction analyses.
Anatomical location and functional constraints (Molinaro et al. [23]; Mendoza Mireles et al. [25]; Karschnia et al. [18])	EOR is constrained by tumor location, involvement of eloquent structures, tumor size, and anticipated neurological morbidity.	A larger RV may reflect anatomical limitations to safe resection rather than inadequate surgical cytoreduction.	Patients with anatomically favorable tumors may be more likely to achieve high EOR/low RV and may also have better outcomes independently of surgery, producing confounding by indication.	Report tumor location, eloquence, multifocality, preoperative neurological status, tumor size, and intended surgical goal; incorporate these variables into adjusted analyses.
Confounding by indication and treatment heterogeneity (Molinaro et al. [23]; Mendoza Mireles et al. [25]; Bette et al. [5])	Patients undergoing more extensive resection may differ systematically in tumor accessibility, performance status, neurological function, and treatment fitness.	Greater RV may similarly identify patients with more challenging tumors or poorer baseline characteristics.	Survival differences may partly reflect underlying patient or tumor characteristics, postoperative morbidity, or differences in adjuvant treatment rather than residual tumor burden itself.	Use multivariable or propensity-based approaches and report radiotherapy, temozolomide, treatment completion, postoperative neurological status, and other relevant treatment variables.
Statistical methodology and threshold selection (Chaichana et al. [4]; Sanai et al. [15]; Brown et al. [16]; Jusue-Torres et al. [17]; Xing and Wang [26]; Woo et al. [27])	Studies differ in EOR categories, thresholds, continuous versus categorical analyses, and covariate selection.	RV studies use different absolute thresholds and may analyze RV continuously or categorically.	Data-driven thresholds, inconsistent adjustment, and different statistical models can yield apparently contradictory conclusions from otherwise similar cohorts.	Prefer prespecified continuous analyses where appropriate; report hazard ratios with 95% CIs, threshold-derivation methods, sensitivity analyses, missing-data handling, and validation where applicable.

Taken together, these factors indicate that EOR and RV should not be viewed as competing measures whose differing results necessarily imply contradictory biological conclusions. Rather, they quantify related aspects of postoperative tumor burden through different mathematical and methodological constructs. When imaging timing, tumor compartment, segmentation, molecular classification, patient selection, and treatment are inconsistent, studies may appear to disagree even when their underlying observations are compatible. The appropriate interpretation is therefore to regard EOR and RV as complementary measures whose prognostic performance depends on standardized measurement and adequate adjustment for clinical and biological context. This provides the rationale for moving from isolated EOR or RV thresholds toward a standardized reporting approach that allows postoperative tumor burden to be interpreted consistently across studies.

Proposed standardized reporting framework for EOR and RV studies

Rationale and Development of the Framework

The preceding sections demonstrate that interpretation of postoperative tumor burden is limited not primarily by the absence of prognostic evidence but by incomplete and inconsistent reporting of the variables required to interpret that evidence. We therefore propose a standardized reporting framework specifically for studies evaluating EOR and RV in GBM. The framework was developed through a structured narrative synthesis of the recurrent methodological limitations identified across the literature reviewed in this article, supplemented by established principles of transparent reporting for observational, prognostic, and clinical studies [41-44]. This author-derived framework was not developed through a formal Delphi process, multidisciplinary consensus meeting, or prospective stakeholder consultation. Accordingly, it should be regarded as a proposed reporting tool rather than a validated reporting guideline. EQUATOR guidance emphasizes that formal reporting guidelines should include an explicit development methodology and broad stakeholder participation, often through iterative Delphi processes and consensus meetings [36].

The framework is intended as a disease- and procedure-specific complement to established reporting standards, not a replacement. STROBE should remain the principal reporting standard for observational cohorts, CONSORT for randomized trials, and TRIPOD or other appropriate prognostic-model guidance when prediction models are developed [42-44].

Core Reporting Domains

The proposed framework adds GBM-specific information needed to determine whether EOR and RV measurements are comparable and clinically interpretable. Table 4 consolidates these requirements into 10 reporting domains.

**Table 4:** Proposed standardized reporting framework for studies evaluating EOR and RV in glioblastoma AI: artificial intelligence, CE: contrast-enhancing, CI: confidence interval, EOR: extent of resection, GBM: glioblastoma, IDH: isocitrate dehydrogenase, KPS: Karnofsky Performance Status, MGMT: O⁶-methylguanine-DNA methyltransferase, MRI: magnetic resonance imaging, OS: overall survival, PFS: progression-free survival, RANO: Response Assessment in Neuro-Oncology, REMARK: Reporting Recommendations for Tumor Marker Prognostic Studies, RV: residual tumor volume, STROBE: Strengthening the Reporting of Observational Studies in Epidemiology, TRIPOD: Transparent Reporting of a Multivariable Prediction Model for Individual Prognosis or Diagnosis, WHO: World Health Organization

Reporting domain	Minimum reporting elements	Rationale
1. Study population and surgical intent	Diagnostic criteria and WHO classification; IDH status; inclusion/exclusion criteria; newly diagnosed versus recurrent disease; biopsy versus intended resection; date range and treatment era relative to adoption of temozolomide chemoradiotherapy (pre- versus post-2005), including the proportion of patients receiving the Stupp regimen; patient flow (number screened, excluded, and analyzed, with reasons for exclusion), including perioperative deaths (e.g., 30-day mortality) and patients not receiving adjuvant therapy, with reasons; definition of time zero (date of surgery preferred).	Mixing biopsy-intent and resection-intent populations can substantially distort EOR distributions and survival comparisons. Molecular classification is essential for interpreting contemporary GBM cohorts. Transparent reporting of patient flow and time zero allows readers to judge whether exclusion of patients who died perioperatively, developed severe deficits, or did not receive adjuvant therapy could bias survival estimates [1,27,34].
2. Patient and tumor characteristics	Age, sex, KPS/performance status (assessed using the same scale as in the postoperative assessment), tumor location, laterality, eloquence/involvement of critical structures, preoperative tumor volume, multifocality, and relevant molecular markers including MGMT promoter methylation.	These variables influence both achievable resection and survival and should be available to assess confounding and case-mix differences [35,45].
3. Surgical approach, feasibility, and adjuncts	Surgical objective; maximal safe versus planned subtotal resection; surgeon/center experience or case volume where available; awake mapping, neuromonitoring, 5-ALA fluorescence, intraoperative MRI, ultrasound, navigation, or other adjuncts.	Surgical technique and institutional or surgeon-level experience can influence achievable EOR, morbidity, and postoperative management. Reporting these factors improves interpretation of differences between cohorts [45-46].
4. Imaging acquisition and timing	MRI field strength; relevant sequences; contrast administration; preoperative imaging interval; postoperative MRI timing, preferably within 24-48 hours and no later than 72 hours when feasible; handling of delayed imaging; methods used to distinguish residual tumor from reactive enhancement, blood products, and ischemia (e.g., T1 subtraction, DWI, comparison with early postoperative or follow-up imaging).	Postoperative enhancement, blood products, ischemia, and other surgical changes can influence apparent residual disease. Imaging timing should therefore be explicitly reported and justified [30,32].
5. Tumor segmentation methodology	Manual, semi-automated, or automated segmentation; software/platform; segmentation protocol; postoperative imaging technique and timing; CE versus non-CE/T2-FLAIR compartment; handling of postoperative cavity and artifacts; number and expertise of readers; interobserver agreement and measurement reliability where available.	Segmentation methodology directly affects measured EOR and RV. Automated approaches may improve reproducibility but require specification of the algorithm and validation context [28,31].
6. Definition and calculation of EOR/RV	Preoperative and postoperative volumes; formula used for EOR; absolute RV in cm³; CE and non-CE components where applicable; categorical versus continuous analysis; assessment of nonlinear associations where appropriate; explicit cutoff definitions, derivation, and validation; and consideration of baseline tumor volume when interpreting EOR thresholds; measurement uncertainty near small-volume thresholds.	EOR terminology and thresholds remain highly heterogeneous. Reporting both percentage EOR and absolute RV facilitates cross-study comparison and prevents apparently similar categories from being treated as equivalent [9,32].
7. Molecular and classification framework	WHO molecular classification, IDH status, MGMT promoter methylation, TERT promoter mutation status where available, and the methods used to determine these biomarkers. Report the distribution of molecular characteristics across resection categories and undertake prespecified molecularly stratified and interaction analyses where sample size permits. Distinguish prognostic associations from evidence of treatment-effect modification.	Molecular reclassification can alter cohort composition and prognosis. Explicit reporting permits comparison between historical and contemporary GBM populations [1,23,44].
8. Confounding and treatment adjustment	Prespecified adjustment for age, KPS, tumor size/location, molecular status, and surgical intent. Postoperative deficits and treatment receipt should be recognized as potential mediators; report primary analyses without mediator adjustment and, where appropriate, separate mediator-adjusted or landmark analyses. Report missing data and methods used to address missingness.	EOR and RV are strongly affected by case selection and treatment pathways. Transparent adjustment is necessary to distinguish the prognostic contribution of residual disease from confounding by indication [35,36].
9. Statistical and prognostic reporting	Kaplan-Meier estimates where appropriate; multivariable Cox models; hazard ratios with 95% CIs; proportional-hazards assessment; continuous and/or prespecified categorical analyses; threshold derivation and validation; sensitivity analyses; missing-data methods; internal/external validation for prediction models.	Transparent statistical reporting reduces selective thresholding and allows independent assessment of prognostic effects. Prognostic studies should follow appropriate reporting principles such as TRIPOD and REMARK where applicable [42,47].
10. Outcomes and functional consequences	OS and PFS with effect estimates; postoperative functional status with KPS and mRS as core measures (NANO or NIHSS optional) at prespecified timepoints (baseline, early postoperative or discharge, and before adjuvant therapy), distinguishing new versus worsened and transient versus permanent deficits; time from surgery to start of radiotherapy/temozolomide; receipt and completion of adjuvant therapy with reasons for non-receipt; neurocognitive outcomes and patient-reported QoL when available; follow-up duration; progression definition.	Survival alone may not capture the consequences of aggressive resection. Functional and patient-reported outcomes provide complementary information when evaluating the clinical meaning of maximal cytoreduction [48].

Application to Existing EOR and RV Literature

Applying this framework to the landmark literature reviewed above shows why apparently comparable studies can yield different conclusions. Lacroix and Sanai established the prognostic association between greater resection and survival but used earlier EOR definitions that differ from subsequent volumetric RANO resect categories [3,15]. Chaichana, Bette, Xing, and Woo later emphasized absolute RV, but their thresholds and volumetric approaches did not match [4-5,26-27]. The RANO resect studies substantially improved standardization by incorporating absolute residual CE volume and defined categories. Yet, subsequent work has demonstrated that postoperative imaging, segmentation, molecular classification, and neurological status remain important sources of heterogeneity [32,35]. Thus, the framework does not imply that earlier studies were methodologically inadequate; rather, it identifies the information needed to interpret their findings more precisely in contemporary evidence synthesis.

Distinguishing Surgical Metrics From Surgical Context

An important practical distinction is that the framework separates the definition of the surgical metric from the broader reporting of the surgical procedure. EOR and RV should be reported together as quantitative postoperative measures. In contrast, the surgical approach, operative intent, feasibility constraints, adjuncts, and surgeon/center characteristics describe the context in which those measurements were achieved. This distinction avoids duplicating information across checklist domains while preserving variables essential for understanding selection and confounding.

Relationship to Existing Reporting Standards and Future Validation

The framework should also be viewed as complementary to existing reporting standards. STROBE provides the general structure for transparent reporting of observational studies, while CONSORT addresses randomized trials and TRIPOD addresses prediction models [42-44]. The proposed framework adds procedure- and disease-specific reporting requirements that those generic standards do not detail sufficiently, particularly postoperative MRI timing, tumor compartment, volumetric segmentation, EOR/RV thresholds, and surgical adjuncts. It is therefore best regarded as a potential GBM-specific reporting extension rather than a competing reporting system.

Finally, the framework is deliberately presented as a starting point for future validation rather than as a completed reporting guideline. No formal Delphi consensus, external stakeholder review, prospective piloting, or interrater reliability testing has yet been performed. Its next stage of development should therefore involve independent application to representative EOR/RV studies by neurosurgeons, neuro-oncologists, neuroradiologists, imaging scientists, statisticians, and methodological experts, followed by assessment of item clarity, reproducibility, feasibility, and association with study interpretability. Such a process could ultimately determine whether the proposed framework warrants formal development as a reporting-guideline extension and potential submission to the EQUATOR Network [36].

Artificial intelligence and large language models in standardizing EOR/RV assessment

Artificial intelligence (AI) may address an important practical limitation identified throughout this review: EOR and RV depend on labor-intensive, observer-dependent tumor segmentation. Importantly, evidence specifically addressing the postoperative setting is less mature than the extensive literature on preoperative glioma segmentation. Helland et al. evaluated deep neural networks for residual tumor segmentation on early postoperative MRI across 956 patients from 12 hospitals and reported a best Dice score of approximately 0.61, with balanced accuracy of approximately 80% for distinguishing residual tumor from gross-total resection; performance was comparable with expert-rater agreement and demonstrated cross-center generalizability [49]. However, AI-generated contours may systematically underestimate RV and should therefore be considered an initial segmentation requiring expert review and correction rather than a replacement for expert assessment. Luque et al. subsequently developed a U-Net-based model specifically for early postoperative MRI and reported a mean external-test Dice score of 0.52 ± 0.03 for residual CE tumor. Although the model underestimated tumor volume in the external cohort, it achieved 0.90 precision and 0.87 recall for maximal versus submaximal EOR classification, and model-derived EOR stratification provided prognostic discrimination comparable with clinician assessment [31]. A further 2024 study using nnU-Net reported median Dice scores of 0.81 for CE tumor and 0.81 for surgical cavities, with 84% accuracy for EOR classification in an independent cohort, supporting the feasibility of automated postoperative assessment while also showing that segmentation performance and downstream EOR classification accuracy are distinct endpoints [50].

These findings are encouraging but do not establish that AI-derived RV can yet replace expert volumetric assessment. Postoperative MRI presents specific failure modes, including blood products, surgical cavity changes, reactive enhancement, small RV, and poorly demarcated non-enhancing or infiltrative disease. Luque et al. specifically observed systematic underestimation of RV in their external dataset despite strong EOR classification performance, illustrating why a model that accurately assigns a categorical EOR class may not necessarily provide sufficiently accurate absolute RV for prognostic threshold analysis [31]. Domain shift across institutions, scanners, acquisition protocols, and patient populations is another concern. Accordingly, AI studies intended for EOR/RV standardization should report the imaging protocol, training and testing populations, reference-standard annotations, model architecture/version, segmentation metrics, volume error, downstream EOR/RV performance, and external-testing results. These requirements are consistent with the 2024 Checklist for Artificial Intelligence in Medical Imaging (CLAIM), which emphasizes transparent reporting of data sources, image acquisition, model development, testing, and reproducibility [51].

Large language models (LLMs) represent a separate and more preliminary opportunity. Unlike image-segmentation models, LLMs should not currently be presented as validated tools for measuring postoperative tumor volume. Their potential role is instead in structured extraction and documentation: converting operative and radiology reports into standardized EOR/RV fields, identifying missing reporting elements, and helping construct structured datasets for retrospective research. Evidence from radiology more broadly suggests that LLMs can facilitate structured reporting, but current studies remain limited and report concerns regarding factual errors, hallucination, privacy, and clinical reliability [52-54]. Therefore, LLM-based documentation should be considered a future research direction rather than an established method of EOR/RV assessment. Recent expert consensus on reporting LLM studies in radiology further emphasizes the need to explicitly report the model, prompting, performance, transparency, risks, and limitations [54].

Before using LLM-extracted EOR/RV data in retrospective survival analyses, validate their accuracy against dual independent human abstraction, with a senior reviewer adjudicating discrepancies. Studies should report field-level accuracy, sensitivity, specificity, and F1-score, together with agreement for continuous measurements such as RV and EOR using appropriate measures such as intraclass correlation. Validation should also assess missing or incorrectly extracted fields and clinically important extraction errors.

AI therefore has the greatest immediate relevance to this framework when used to improve segmentation and quantitative measurement reproducibility. At the same time, LLMs may eventually support the reporting and data-standardization layer. In practical terms, studies using AI-assisted EOR/RV assessment should document the model, software version, imaging inputs, reference standard, testing performance, and clinically relevant failure cases alongside the conventional clinical and imaging variables specified in the reporting framework. This approach would make AI a transparent component of the measurement process rather than an unexplained source of additional variability.

Limitations

This review has several limitations. First, it is a narrative and critical appraisal rather than a systematic review or meta-analysis; therefore, the literature was synthesized qualitatively, and selection and interpretation bias cannot be excluded. The review does not provide a formal quantitative estimate of the comparative prognostic performance of EOR and RV, and the included studies differ substantially in patient populations, imaging protocols, definitions, and statistical methods.

Second, the proposed standardized reporting framework is author-derived and based on recurrent methodological limitations identified across the reviewed literature. It was not developed through a formal Delphi process, multidisciplinary consensus meeting, prospective pilot study, or external stakeholder validation. Accordingly, the framework should be regarded as a proposed starting point rather than a validated reporting instrument. Its individual domains and reporting requirements require prospective evaluation for clarity, feasibility, reproducibility, and clinical relevance before any formal adoption.

Third, much of the available evidence concerning EOR and RV is retrospective and observational. Consequently, confounding by indication cannot be eliminated. Patients undergoing more extensive resection may differ systematically from those with greater residual disease with respect to tumor location, size, neurological status, molecular characteristics, surgical feasibility, surgeon or institutional expertise, and receipt or completion of adjuvant therapy. Even appropriately adjusted analyses may therefore not establish a causal relationship between a particular EOR or RV threshold and survival.

The search period (2000-2026) spans the introduction of temozolomide chemoradiotherapy, and cohorts from earlier and later eras differ in adjuvant treatment and molecular classification. Additionally, studies that exclude or censor patients with early death, severe postoperative deficits, or failure to complete adjuvant therapy may overestimate the benefit of radical resection. The proposed framework improves the transparency of such exclusions but cannot correct for them in retrospective data.

Additionally, postoperative imaging timing was not uniform across the studies discussed and was not an eligibility criterion; reported RV and EOR values may therefore not be directly comparable, and reactive enhancement could not be excluded retrospectively.

Finally, contemporary AI-assisted segmentation and LLM applications remain evolving technologies. Although early postoperative segmentation studies demonstrate encouraging reproducibility and classification performance, external validation across institutions, scanners, imaging protocols, and heterogeneous postoperative appearances remains limited. Similarly, the proposed applications of LLMs to structured reporting and data extraction are preliminary and should not be considered validated methods for measuring postoperative tumor burden. These limitations reinforce the need for future prospective, multicenter studies using standardized imaging, molecular classification, quantitative volumetry, functional outcomes, and transparent statistical methodology to validate both the prognostic measures and the proposed reporting framework.

## Conclusions

EOR and RV are important prognostic measures in GBM, but heterogeneity in definitions, imaging protocols, segmentation methods, tumor compartments, thresholds, and adjustment for clinical and molecular factors substantially limits their interpretation. The apparent discordance between EOR- and RV-based studies should therefore not necessarily be interpreted as biological disagreement but may reflect differences in how postoperative tumor burden is measured and reported. Contemporary RANO resect classifications represent an important advance toward standardization, but additional methodological transparency is required to facilitate meaningful cross-study comparison.

This review proposes a practical, disease-specific reporting framework encompassing patient and tumor characteristics, surgical context, imaging, segmentation, EOR/RV definitions, molecular classification, confounder adjustment, statistical methodology, and patient-centered outcomes. The framework is not yet validated and should be considered a starting point for future multidisciplinary consensus and prospective testing. Standardized reporting, complemented by reproducible volumetric assessment and emerging AI-based tools, may strengthen evidence synthesis and improve the interpretability of surgical outcome studies in GBM.

## Appendices

**Table 5:** Risk of bias assessment of prognostic studies using the QUIPS tool * Overall risk is a qualitative synthesis of the six QUIPS domains rather than a numerical summation. QUIPS: Quality In Prognosis Studies

Study	Study participation	Study attrition	Prognostic factor measurement	Outcome measurement	Study confounding	Statistical analysis & reporting	Overall*
Lacroix et al., 2001 [3]	Moderate	Moderate	Moderate	Low	Moderate	Moderate	Moderate
Chaichana et al., 2014 [4]	Moderate	Moderate	Low	Low	Moderate	Moderate	Moderate
Bette et al., 2018 [5]	High	Moderate	Low	Low	Moderate	Moderate	High
Antony et al., 2026 [10]	Moderate	Moderate	Low	Low	Moderate	Moderate	Moderate
Sanai et al., 2011 [15]	Low	Low	Moderate	Low	Moderate	Moderate	Moderate
Karschnia et al., 2023 [18]	Low	Moderate	Low	Low	Low	Low	Moderate
Molinaro et al., 2020 [23]	Low	Low	Low	Low	Low	Low	Low
Mendoza Mireles et al., 2026 [25]	Low	Low	Low	Low	Moderate	Low	Moderate
Xing & Wang, 2018 [26]	Moderate	Moderate	Moderate	Low	Moderate	Moderate	Moderate
Woo et al., 2019 [27]	Low	Moderate	Moderate	Low	Moderate	Moderate	Moderate
Ellingson et al., 2018 [28]	Moderate	Moderate	Low	Low	Moderate	Low	Moderate
Filice et al., 2026 [29]	Moderate	Moderate	Low	Low	Moderate	Low	Moderate
